# Pulmonary Toxicity and Adjuvant Effect of Di-(2-exylhexyl) Phthalate in Ovalbumin-Immunized BALB/c Mice

**DOI:** 10.1371/journal.pone.0039008

**Published:** 2012-06-12

**Authors:** Jing Guo, Bing Han, Longjuan Qin, Bing Li, Huihui You, Jiwen Yang, Dandan Liu, Chenxi Wei, Eewa Nanberg, Carl-Gustaf Bornehag, Xu Yang

**Affiliations:** 1 Hubei Key Laboratory of Genetic Regulation and Integrative Biology, Huazhong Normal University, Wuhan, China; 2 Department of Chemistry and Biomedical Science, Karlstad University, Karlstad, Sweden; 3 Public Health Sciences, Department of Health and Environment, Karlstad University, Karlstad, Sweden; Ludwig-Maximilians-University Munich, Germany

## Abstract

**Background:**

Asthma is a complex pulmonary inflammatory disease, which is characterized by airway hyperresponsiveness, variable airflow obstruction and inflammation in the airways. The majority of asthma is allergic asthma, which is a disease caused by type I hypersensitivity mediated by IgE. Exposures to a number of environmental chemicals are suspected to lead to asthma, one such pollutant is di-(2-ethylheyl) phthalate (DEHP). DEHP is a manufactured chemical that is commonly added in plastic products to make them flexible. Epidemiological studies have revealed a positive association between DEHP exposure and asthma prevalence.

**Methodology/Principal Findings:**

The present study was aimed to determine the underlying role of DEHP exposure in airway reactivity, especially when combined with allergen exposure. The biomarkers include pulmonary histopathology, airway hyperresponsiveness (lung function), IgE, IL-4, IFN-γ and eosinophils. Healthy balb/c mice were randomly divided into eight exposure groups (n = 8 each): (1) saline control, (2) 30 µg/(kg•d) DEHP, (3) 300 µg/(kg•d) DEHP, (4) 3000 µg/(kg•d) DEHP, and (5) ovalbumin (OVA)-sensitized group, (6) OVA-combined with 30 µg/(kg•d) DEHP, (7) OVA-combined with 300 µg/(kg•d) DEHP, and (8) OVA-combined with 3000 µg/(kg•d) DEHP. Experimental tests were conducted after 52-day DEHP exposure and subsequently one week of challenge with aerosolized OVA. The principal findings include: (1) Strong postive associations exist between OVA-combined DEHP exposure and serum total IgE (T-IgE), as well as histological findings. These positive associations show a dose-dependent low dose sensitive effect of DEHP. (2) IL-4, eosinophil recruitment and lung function are also indicators for adjuvant effect of DEHP.

**Conclusions/Significance:**

Our results suggest that except the significant changes of immunological and inflammatory biomarkers (T-IgE, IL-4, IFN-γ and eosinophils), the pulmonary histological (histopathological examination) and physiological (lung function) data also support that DEHP may promote and aggravate allergic asthma by adjuvant effect.

## Introduction

Numerous studies have suggested that the notable increase in allergic subjects seen over the last decades is due to environmental changes occurring throughout the world [1], [2]. It has been suggested that exposure to specific chemical pollutants in indoor air and dust may be associated with the development of respiratory problems [3]–[7]. Among these chemicals, Di-(2-ethylhexyl) phthalate (DEHP), a commonly used plasticizer in soft poly vinyl chloride (PVC) materials, is attracting more health concern. ATSDR and EPA have reported on adverse reproductive effects in which the daily intake range of DEHP for an adult was estimated to be 3–30 µg/kg/d [8], and the exposure assessment of NTP-CERHR was up to 30 µg/kg/d [9]. Generally, children are thought to be more susceptible since they are more likely to be exposed to DEHP. ATSDR reported that the daily exposure of children to DEHP by sucking or chewing on toys or other articles was up to 85 µg/kg/day [8]. And the maximum exposure even exceeded 100 µg/kg/day in some cases. In addition, humans can be exposed to DEHP during certain medical procedures which are likely to cause higher exposure than the common everyday routes. The source of oral exposure to DEHP in the medical device context includes enteral feeding bags, nasogastric tubing, and denture materials. In this special case, the estimated amount of DEHP received by highly exposed individuals from enteral nutrition storage bags is up to around 2 mg/day. However, the total amount of DEHP medical oral exposure is unclear [10].

Epidemiological studies have offered evidence for the possible relationship between exposure to phthalates and PVC materials and the risk of asthma and allergy both in workers of plastic industry and in children [11]–[16]. Some of these studies have suggested a higher risk of respiratory problems among those exposed in occupational as well as residential environments. Several animal studies have indicated that co-administration of phthalates modulate the immune response to antigen although the biological mechanism remains unsolved. In one study, DEHP was showed to provoke a significant increase in the production of IgG1 in a mice model with subcutaneous administration of a model antigen, ovalbumin (OVA) [17]. In similar studies, MEHP, one metabolite of DEHP, was found to cause an increase in IgE levels [18], [19]. More recent studies were performed in murine models mimicking human exposure pathways. Larsen et al. used a long-term inhalation model with DEHP and reported modulating effect on IgG1 response as well as inflammatory markers [20]. Ourselves have reported on DEHP promoting inflammatory responses in DEHP treated OVA-immunized rats [21]. However, in the studies by Larsen et al [17], [18] immunosuppression was also seen after injection of high doses of MEHP, not DEHP. And Larsen and Nielsen reported that extended treatment with DEHP desensitized OVA-induced immune responses [22]. On the other hand, there are reports on no antibody response including production of IgE or Th2 cytokines in DEHP exposed mice but in those studies topical application of phthalates was used [23], [24]. Consequently, the relevance of DEHP exposure on the development of allergic asthma is still a controversy.

In addition, most of the previous studies were carried out applying short term exposure regimes and the models did not exhibit typical human asthma like properties such as airway hyperresponsiveness (lung function), histopathological examination for lung inflammation (lung tissue cell infiltration) and airway remodeling and/or airway mucus. We, therefore, studied in greater detail the suspected impact of DEHP on airway reactivity related to allergic asthma by using a modified long-term oral exposure OVA-sensitized murine model. This study aimed to address the effect of long-term DEHP exposure on airway inflammation and allergic response to OVA related to human asthma, and to establish a representative experimental platform for further studies on the molecular mechanisms of DEHP-induced asthma.

## Materials and Methods

### Ethics Statement

The experimental procedures were approved by the Office of Scientific Research Management of Central China Normal University, with the certification on Application for the Use of Animals dated March 26, 2010 (approval ID: CCNU-SKY-2010-005). According to the requirements of ethics only 8 mice were used in each treatment group to minimize the number of experimental animals and to ensure the validity of statistical power.

### Animals

Balb/c mice (males, 5–6 weeks old and 17–19 g) were purchased from the Hubei experimental animal center (Wuhan China) and housed in cages with pinewood sawdust bedding. They were maintained in a pathogen-free room at temperatures between 20–25°C and 50–70% humidity. OVA-free food and tap water were used. The mice were quarantined for at least 7 days before study initiation.

### Exposure and immunization protocol

The first and the second set of mice were sequentially treated with the same exposure and immunization protocol. Each set of 64 balc/c mice were randomly divided into eight exposure groups: (1) saline control (saline control), (2) 30 µg/(kg•d) DEHP (DEHP30), (3) 300 µg/(kg•d) DEHP (DEHP300), (4) 3000 µg/(kg•d) DEHP (DEHP3000), (5) OVA-sensitized group (OVA only), (6) OVA-combined with 30 µg/(kg•d) DEHP (OVA+DEHP30), (7) OVA-combined with 300 µg/(kg•d) DEHP (OVA+DEHP300), and (8) OVA-combined with 3000 µg/(kg•d) DEHP (OVA+DEHP3000).

**Figure 1:**
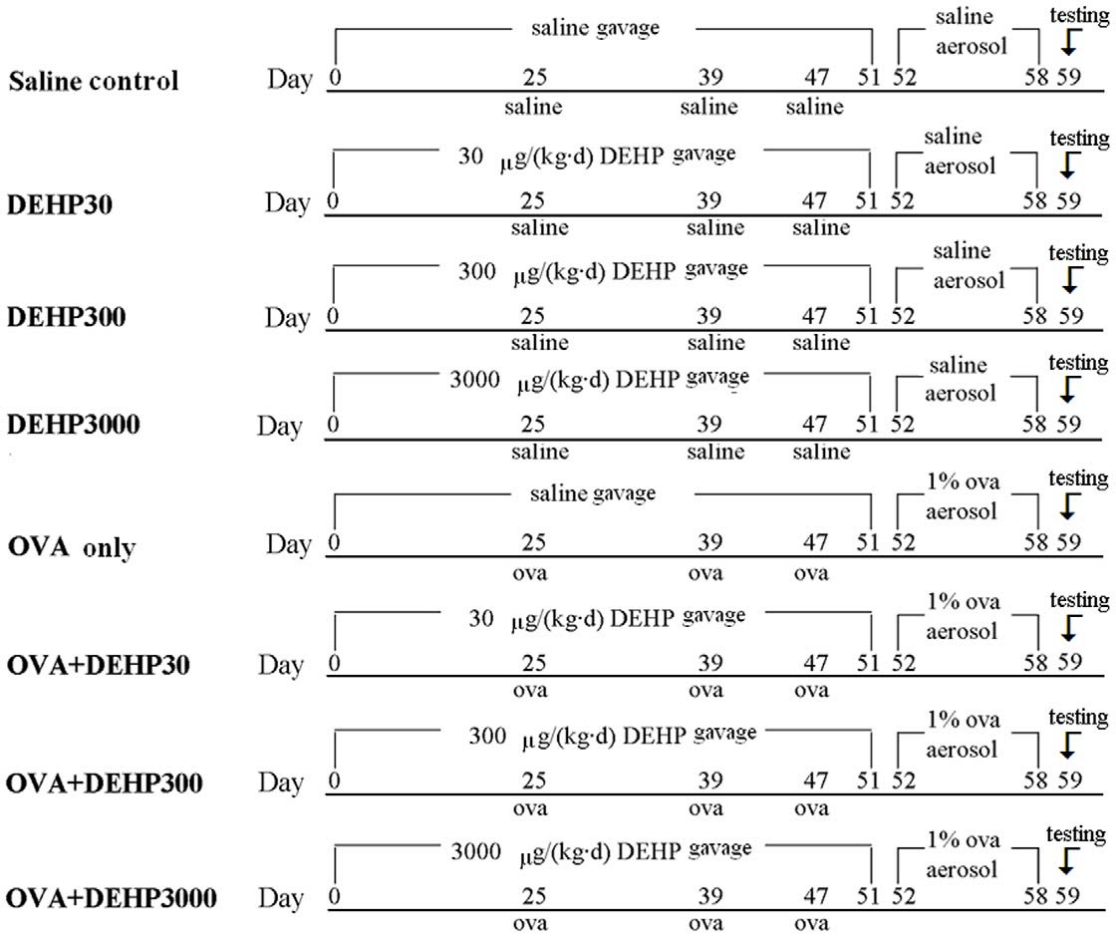
Exposure and immunization schedule.

The mice were gavaged with DEHP or saline from day 0 to 51 (52 times), and were sensitized with OVA+ Al(OH)_3_ (20 µg OVA and 1.75 mg Al(OH)_3_ in 300 µl saline each time) or saline (300 µl saline each time) by subcutaneous injection on day 25, 39 and 47. This was then followed by an aerosol challenge in 1% OVA (30 min/d) from days 52 to 58 (7 times) using an ultrasonic nebulizer (Yuyue 402A type I, China). Before administration DEHP was dissolved in Tween 80 (CAS No. 9005-65-6, analytical reagent, Sinopharm Chemical Reagent Co, Ltd. China) (DEHP: Tween 80 is 1∶1 in w/w) together with saline, and then add more saline to the required volume. All of the DEHP dissolved very well. The detailed protocol outline is shown in Fig. 1.

To prevent adverse effects on measures of lung cytokines/pulmonary architecture due to influence from the methacholine used in airway hyper-responsiveness (AHR; see below) assessments, dedicated groups of mice (for each of the above regimens) were employed. One set of 64 mice was treated for 59 days according to the above-regimen(s) and then used for AHR tests and BALF sample collection; a second set of 64 mice was treated as above and then used directly (i.e., non-lavaged) for cytokine (left lung) and histopathologic (right lung) analyses.

### Measurement of airway responsiveness

For the first set of 64 balb/c mice airway hyperresponsiveness as an indicator of lung function was tested 24 hours after the final aerosol exposure using the AniRes 2005 lung function system (Bestlab, AniRes 2005, version 2.0, China). Animals were anesthetized by intraperitoneal injection with pentobarbital sodium (Urchen) at a dose of 95 mg/kg and were then connected to a computer-controlled small animal ventilator via a tracheal cannula. The respiratory rate was preset at 95 times per min and the time ratio of expiration/inspiration was 1.5∶1. When the parameters were stable, an injector needle was inserted into the jugular vein and methacholine (O-Acetyl-β-methylcholine chloride, MCh, Sigma-Aldrich) was administered through a catheter with increased dosages of 0.025, 0.05, 0.1, and 0.2 mg/kg every 5 min. Mouse airway responsiveness was examined by expiratory resistance (Re), inspiratory resistance (Ri) and dynamic lung compliance (Cldyn). The relative area (R-area) was defined as the area under the peak curve of Re or Ri and beyond the baseline level during the 250 s time frame. The distance from the wave trough to the baseline level of the Cldyn curve was adopted to quantitatively assess lung compliance [25].

### Counting of eosinophils and total cells in BALF

BALF was collected after airway hyperresponsiveness measurements. After the final 0.2 mg/kg MCh challenges, the mice were removed from the operation box. Using a 1 ml syringe, 0.5 ml aliquots of 0.9% NaCl solution was injected into the trachea and lungs through the tracheal cannula, the chests of mice were gently massaged and rinsed for approximately 1 min, and then the liquid was withdrawn with the same syringe. The process was repeated 3 times, and the total quantity of BALF was 1.2 ml–1.3 ml for each sample. The cellular fraction from BALF was collected by centrifugation at 1500 rpm (200× g) for 10 min at 4°C and resuspended in 1 ml saline. Total cell counts were obtained from 500 µl of the cell suspension using a blood cell analysis system (Sysmex KX-21, Japan). Another 500 µl cell suspension was prepared for eosinophil counts. Eosinophils were stained using Eosin Y sodium solution (Amresco, high purity grade, USA), and cell numbers were assessed using a hemocytometer (Sigma-Aldrich, USA) and a microscope (Olympus). Neutrophils, macrophages or monocytes were not counted because of the limited amount of BALF.

The relative number of eosinophils to total cell number in BALF was used in this study as an indicator for eosinophil recruitment, because it overcomes the inconsistency of BALF sample volume between individual mice and is thus comparable within the study. Relative number of eosinophils = eosinophil number in BALF/total cell number in BALF.

### Histological examination

Following BALF collection, the lungs were isolated for histopathology slice preparation. All the samples were incubated in fixation solution (saturated 2, 4, 6-trinitrophenol∶formalin∶glacial acetic acid = 15∶5∶1) for 24 hours at room temperature and then cut into pieces, and stained with hematoxylin and eosin (H&E Sigma-Aldrich, USA). The stained pieces were then embedded in paraffin, sectioned into 10 µm slices and observed under a microscope (Leica DM 4000B, Germany).

### Detection of serum immunoglobulin E

For the second set of 64 balb/c mice blood was drawn after finishing of the exposure and immunization treatments, and serum samples were collected after centrifuging the blood (2400 rpm, i.e. 500×g, 4°C, 10 min) and stored at −80°C until analyzed. The amounts of total IgE and anti-OVA-IgE in the serum from different groups of mice were determined using the total IgE ELISA kit (Biolegend, USA). The sensitivities are 15 pg/ml for both ELISA kits of total IgE and anti-OVA-IgE. Since the anti-OVA-IgE standards were not available, the levels of anti-OVA-IgE could just be indicated as absorbance (492 nm) but not as values of ng/ml.

### Detection of the cytokines in lung tissue

After serum sample collection, the lungs were excised and homogenized in 0.9% NaCl solution, and fractionation of the soluble fraction was done by centrifugation (1500 rpm, i.e. 200× g, 4°C, 10 min). The tissue levels of IL-4 and IFN-γ were measured by ELISA kits according to the manufacture's instructions (eBioscience, USA). The sensitivities of all ELISA kits are 15 pg/ml.

### Statistical analysis

**Figure 2:**
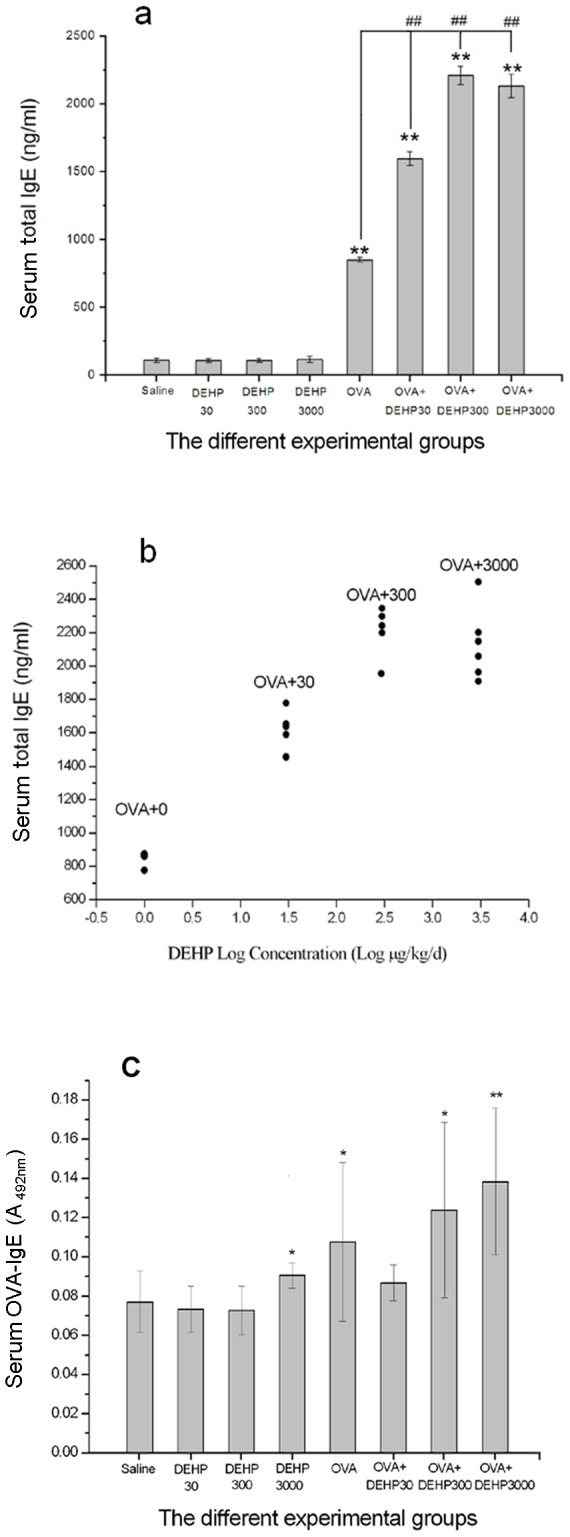
Serum IgE. (a) Total IgE levels in the eight different exposure groups. ** p<0.01, compared with the saline control; ## p<0.01, compared with the OVA only group; (b) Dose-response relationship between DEHP (+OVA) and total serum IgE levels; (c) OVA-IgE levels in the eight different exposure groups. Group data were expressed as means±standard deviation (SD). * p<0.05, ** p<0.01, compared with the saline control.

**Figure 3:**
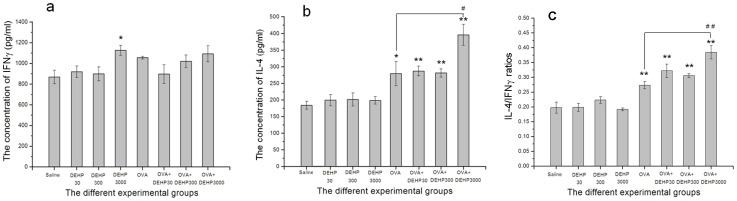
IFN-γ and IL-4 levels in lung tissue. (a) pulmonary tissue IFN-γ levels in each group; (b) pulmonary tissue IL-4 levels in each group; (c) IL-4/IFN-γ ratios in each group. Group data were expressed as means±standard deviation (SD). * p<0.05, ** p<0.01, compared with the saline control; ^#^ p<0.05, ^##^ p<0.01, compared with OVA only group.

**Figure 4:**
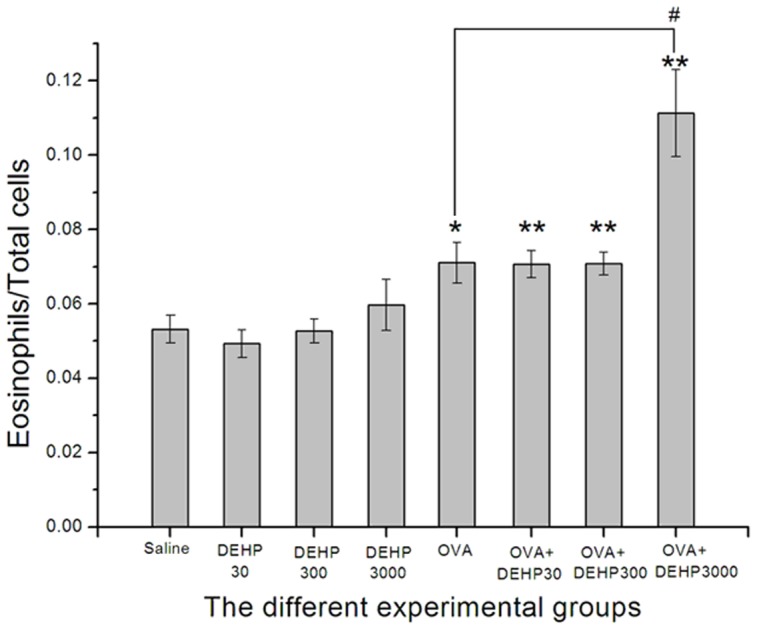
The relative number of eosinophil to total cell numbers in BALF. Group data were expressed as means±standard deviation (SD). * p<0.05 and ** p<0.01, compared with the saline control; ^#^ p<0.05, compared with the OVA only group.

**Figure 5:**
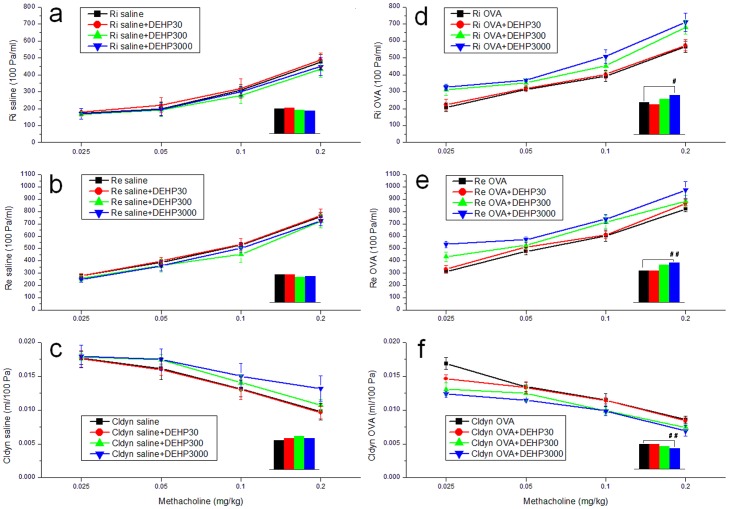
Airway hyperresponsiveness (AHR) measurements in mice. Covariance analyses (SPSS) were used in these tests. (a) Ri values of saline control and DEHP groups; (b) Re values of saline control and DEHP groups; (c) Cldyn values of saline control and DEHP groups; (d) Ri values of OVA only and OVA+DEHP groups; (e) Re values of OVA only and OVA+DEHP groups; (f) Cldyn values of OVA only and OVA+DEHP groups. Group data were expressed as means±standard deviation (SD).

Statistic Program for Social Sciences (SPSS, version 13.0) was used for our data statistic analysis. Covariance analysis was performed for the AHR assessment data, in which the biomarkers (Ri, Re and Cldyn) were taken as dependent variables, DEHP exposure values were taken as fixed factors and MCh doses were taken as covariates, and F and p values were calculated. Data from the other measurements were analyzed using the parametric tests i.e. one-way ANOVA followed by the LSD test. P values less than 0.05 were considered statistically significant and all data were expressed as means±standard deviation (SD) and exhibited in graphs and charts. In figures 2, 3, 4, 5, the statistical test (LSD test for figures 2, 3, 4, t test for figure 5) of two independent samples for OVA only group were done only to OVA+DEHP30, OVA+DEHP300 and OVA+DEHP3000, but not to DEHP30, DEHP300 and DEHP3000, in order to observe if any adjuvant effect existed.

## Results

### Levels of serum IgE

Fig. 2a and b, shows the measurement data of serum total IgE after DEHP-treatment in the absence or presence of OVA-sensitization. Four important findings were revealed: (1) Exposure for only DEHP (DEHP30, DEHP300, DEHP3000) did not cause any changes in serum total IgE (T-IgE), and the T-IgE level for these groups were not different from the saline control group; (2) The serum total IgE levels of all OVA-immunized groups (OVA only, OVA+DEHP30, OVA+DEHP300, OVA+DEHP3000) were significantly inceased in relation to saline and DEHP only exposure groups (saline control, DEHP30, DEHP300, DEHP3000) (p<0.01); (3) From the scatter of Fig. 2b, a dose-response relationship up to OVA+300 but not further between logarithmic concentrations of OVA+DEHP and serum total IgE levels was evident in Fig. 2b; (4) Most important, already the lowest dose of OVA+DEHP30 caused a significant increase in serum T-IgE compared with the OVA only group, which suggests that an adjuvant effect occurs at low levels of DEHP.

From the results of analysis of anti-OVA-IgE in Fig. 2c, we saw that: (1) anti-OVA-IgE (OVA-IgE) did differ between saline and OVA control group; (2) the OVA-IgE levels in the highest DEHP group (DEHP3000) and higher OVA+DEHP exposure groups (OVA+DEHP300, OVA+DEHP3000) had significant differences (p<0.05, p<0.05, p<0.01) compared with the saline group. (3) the OVA-IgE levels in the all OVA+DEHP exposure groups (OVA+DEHP30, OVA+DEHP300, OVA+DEHP3000) had no statistic significant difference compared with the OVA exposure alone group (p>0.05);

### Cytokines in lung tissue

The levels of the Th1 cytokine IFN-γ and the Th2 cytokine IL-4 were assessed in lung tissue samples (Fig. 3). OVA did not affect the levels of IFN-γ and only the highest dose of DEHP with or without OVA increased IFN-γ levels (Fig. 3a). This finding suggests that Th1 cytokines were not induced in this exposure model. In contrast, there were significant increases of IL-4 in the OVA (p<0.05) and OVA+DEHP groups (p<0.01) compared with saline control group (Fig. 3b). The lower doses of OVA+DEHP (OVA+DEHP30 and OVA+DEHP300) did not significantly alter IL-4 levels compared with OVA only group, while OVA+DEHP3000 did cause a significant potentiation (p<0.05). The IL-4 response was even more stringent when IL-4 expression was calculated and expressed related to IFN-γ in the correspondning sample.

### The relative number of eosinophils to total cells in BALF

From figure 4 we can see, that the ratio of eosinophils to total cells in BALF in the OVA-combined with DEHP exposure groups (OVA only, OVA+DEHP30, OVA+DEHP300, OVA+DEHP3000) was significantly increased compared to saline control group, p<0.05 and p<0.01). The DEHP only exposure groups (DEHP30, DEHP300, DEHP3000) showed no significant effects on the ratio of eosinophils to total cells (p>0.05), while the ratio in OVA+DEHP3000 group was increased compared with OVA only group (p<0.05), indicative of an adjuvant effect on eosinophil recruitment.

### Measurement of airway hyperresponsiveness (AHR)

Figure 5 shows the results of measurements of airway responsiveness. (1) In all of the groups, the expiratory and inspiratory resistance increased with the increase of methacholine levels (p<0.01), while the Cldyn decreased (p<0.01). (2) In OVA-sensitized mice (OVA only, OVA+DEHP30, OVA+DEHP300, OVA+DEHP3000), the Ri and Re values were increased compared with the same DEHP doses in saline-treated mice (saline, DEHP30, DEHP300, DEHP300). (3) DEHP exposure had no significant influence on the Ri, Re and Cldyn values (Ri, F = 0.609, p>0.05; Re, F = 1.159, p>0.05; Cldyn, F = 0.445, p>0.05) compared with the saline control, but (4) OVA+DEHP exposure resulted in a significantly enhanced expiratory resistance and inspiratory resistance (Ri, F = 12.804, p<0.01; Re, F = 11.329, p<0.01) compared with OVA only. In the OVA+DEHP3000 group (panel d, e, f.), there is a relatively high resistance and relatively low compliance already at 0.025 mg/kg MCh. This may be due to mucus (airway obstruction) in the airways in addition to constriction of smooth muscles. (5) Further multiple comparisons are summarized in table 1. According to the data, DEHP at the highest exposure level promoted AHR in OVA-sensitized mice, see Fig. 5 and Table 1.

**Table 1:** Multiple covariance analyses for AHR measurements.

Biomarkers (Units)	Saline control group vs DEHP groups							OVA only group vs DEHP+OVA groups						
	Saline	DEHP30		DEHP300		DEHP3000		OVA	DEHP30+OVA		DEHP300+OVA		DEHP3000+OVA	
	mean	mean	VS Saline: P value	mean	VS Saline: P value	mean	VS Saline: P value	mean	mean	VS OVA: P value	mean	VS OVA: P value	mean	VS OVA: P value
Ri (100 Pa/ml)	303.6	313.1	0.825	285.1	0.955	276.5	0.990	386.1	363.9	0.565	429.5	0.101	474.1	0.045
Re (100 Pa/ml)	503.6	500.6	0.827	459.1	0.624	474.5	0.711	575.0	572.6	0.821	676.6	0.064	717.0	0.001
Cldyn (ml/100 Pa)	0.014	0.015	0.536	0.016	0.229	0.015	0.427	0.012	0.012	0.803	0.011	0.208	0.010	0.005

Covariance analyses were used for AHR testing. Dependent variable: biomarkers; Fixed factor: DEHP doses; Covariate: MCh doses.

### Histological examination

**Figure 6:**
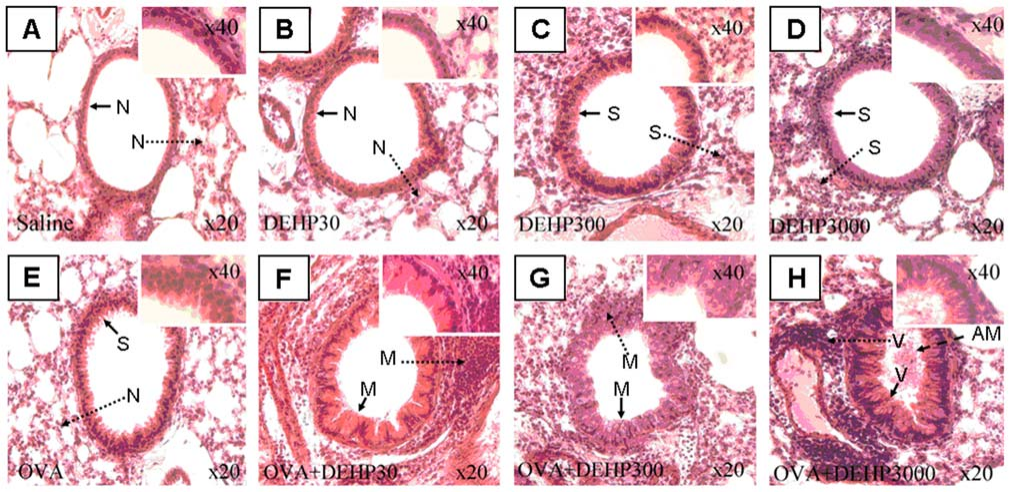
Histopathology of lung tissue with parenchymal inflammation (tissue cell infiltration) and airway remodeling. Lung tissue was fixed, stained with H&E, and sectioned in 10-µm slices. **→**: Bronchial remodeling; **----▸**: lung tissue cell infiltration; **N**: normal conditions; **S**: slight changes; M: moderate changes; V: severe changes. AM **– –** ▸: airway mucus. (A. saline control; B. DEHP30; C. DEHP300; D. DEHP3000; E. OVA only; F. OVA+DEHP30; G. OVA+DEHP300; H. OVA+DEHP3000).

Histological analyses of distal bronchial tissue revealed typical pathological features of asthma-like inflammation and structural alterations in the OVA-immunized groups (Fig. 6, panel E–H) In contrast to the non-sensitized mice, OVA-sensitized animals showed leukocyte infiltration in the surrounding peribronchiolar areas, epithelial folding and thickened subepithelial cell layers (airway remodeling), findings which are normally associated with asthma in humans. There were two key findings as illustrated in figure 6: (1) It was clear that moderate histological changes were found in the OVA+DEHP30 group, whose DEHP exposure dose was relatively low; (2) in the OVA+DEHP3000 group the histopathologic changes were severe, with prominent airway mucosa thickening and epithelial folding and a pronounced airway obstruction, all signs of airway remodeling. Thus a dose-dependent low dose sensitive effect of DEHP was identified on airway tissue reactivity.

### Result summary

The results of this study are summarized in Table 2.

**Table 2:** Summary of the experimental results.

Parameters System for evaluating asthma	DEHP exposure groups				OVA-combined DEHP exposure groups			
	Saline control	DEHP30	DEHP300	DEHP3000	OVA only	OVA+ DEHP 30	OVA+ DEHP 300	OVA+ DEHP 3000
Serum OVA-IgE	–	NS	NS	↑*	–	ns	ns	ns
Th1 cytokine: IFN-γ	–	NS	NS	↑*	–	ns	ns	ns
Th2 cytokine: IL-4	–	NS	NS	NS	–	ns	ns	↑#
IL-4/IFN-γ ratios	–	NS	NS	NS	–	ns	ns	↑##
Eosinophil/Total Cells	–	NS	NS	NS	–	ns	ns	↑#
AHR: Re	–	NS	NS	NS	–	ns	ns	↑##
AHR: Ri	–	NS	NS	NS	–	ns	ns	↑#
AHR: Cldyn	–	NS	NS	NS	–	ns	ns	↑##
Serum total IgE	–	NS	NS	NS	–	↑##	↑##	↑##
Histopathology: Tissue cell infiltration	Normal	Normal	Slight	Slight	Normal	Moderate	Moderate	Severe
Histopathology: Airway remodeling	Normal	Normal	Slight	Slight	Slight	Moderate	Moderate	Severe

**–: As statistical control group; Compared with saline control: NS p>0.05,**

* **p<0.05,**

** **p<0.01; Compared with OVA only group: ns p>0.05,**

# **p<0.05,**

## **p<0.01.**

In the result over-view and data summary presented in Table 2 we have divided the biomarkers (measured parameters) into three groups. The first group is “non-sensitive biomarkers”, include serum OVA-IgE and INF-γ. From a theoretical viewpoint the serum OVA-IgE should be a sensitive biomarker, but this was not confirmed in the present experimental set up. The second group is “moderately sensitive biomarkers”, including IL-4, recruitment of eosinophils and airway hyperresponsiveness (Re, Ri and Cldyn). Our results shows that these biomarkers may indicate an adjuvant effect, but only in the highest DEHP dose exposure group (OVA+DEHP3000). The third group include total serum IgE and histopathological examinations (airway remodeling and lung tissue immune cell infiltration). These biomarkers were more affected by DEHP and thus more strongly indicating an adjuvant effect by DEHP in this model, and our result points to a dose-response relationship.

## Discussion

### The main findings

The main findings of this study include: (1) Strong postive associations exist between OVA-combined DEHP exposure and serum total IgE, as well as histological examination. These positive associations show dose-dependent low dose sensitive effects of DEHP. (2) IL-4, eosinophil recruitment and AHR are also effective indicators for DEHP at somewhat higher concentrations. (3) Like other animal DEHP studies [17], [18], [22], [24], we did not find statistical association between OVA exposure and anti-OVA-IgE. (4) Higher level DEHP exposure alone may cause significantly increased IFN-γ.

### Adjuvant effect of DEHP

Our study suggests, that airway reactivity and immune responses induced by DEHP-OVA co-exposure may be mediated by an adjuvant effect of DEHP. In this study we find that, in the absence of an allergen, OVA, DEHP exposure did not cause obvious histological and physiological changes in the mice; while with co-exposure with OVA, DEHP has the ability to stimulate the body to produce large amounts of IgE, and to cause histological changes such as cell infiltration in lung tissue and airway remodeling at low exposure level; and to induce physiological changes such as pulmonary dysfunction at higher exposure levels. These phenomena can be explained by an adjuvant effect.

Adjuvant effect of DEHP involves two groups of important mechanistic biomarkers: “immune response” and “asthma pathology”. The former include IgE, IgG1, IL-4, IFN-γ, and the latter include lung histopathology and function, eosinophil recruitment. Some studies [17], [20], [22], [23], [24], [26] had just focused on DEHP-induced “immune response”; one other study [21] had just focused on DEHP-induced “asthma pathology”. A literature review has summarized these studies in detail, see table 2 in Bornehag and Nanberg [13]. The present study is the first to our knowledge that combines both groups of biomarkers for understanding the adjuvant effect of DEHP. In this study we have assessed the changes of both two groups of biomarkers, and defined a “DEHP-exposure mouse model”, which is useful for mechanistic research related to human allergic disorder.

So far the molecular mechanism of adjuvant effect of DEHP is not clear. We have speculated that DEHP exposure may up-regulate thymic stromal lymphopoitin (TSLP) protein expression [27], and TSLP was found to up-regulate IgE expression and promote Th2 immune response [28], [29]. More studies are needed for understanding the molecular mechanism on the adjuvant effect of DEHP, in order to effectively prevent related health problems in the future.

### “Conditional risk factor” and “conditional attenuating agent”

The unbalance of Th1 and Th2 immune response is an important pathological basis for allergy and allergic asthma. IFNγ is a key biomarker for Th1, and IgE and IL-4 are key biomarkers for Th2 immune response. In this study we found DEHP exposure alone (Fig. 3a, group DEHP3000) caused an limited but significant (p<0.05) increase in IFN-γ. Our study results suggest that, the biological effects of DEHP are different depending on presence or absence of an antigen (OVA). With OVA co-exposure, DEHP is a strong agent to promote Th2-related immune responses, (allergy and asthma); but without OVA co-exposure, DEHP becomes a weak agent for a Th1 immuno response. This situation is similar to what we found in the adjuvant effect of formaldehyde [30], (see the Fig. 6). Therefor DEHP is a potential “conditional risk factor”.

It is also interesting that DEHP is not only a “conditional risk factor”, but also a “conditional attenuating agent”. In a specially designed long-term low-dose OVA co-exposure model, DEHP was shown to induce OVA-desensitization. In mice [31] find that: compared with the OVA-sensitised control mice, multiple co-exposures to DEHP+OVA reduced the OVA-IgG1 level and reduced the OVA-IgE/OVA-IgG2a ratio. This suggests that DEHP may attenuate long-term allergic sensitisation, as the IgE/IgG2a ratio has been shown to correlate with the degree of anaphylaxis. So, like allergens, DEHP is also of two-direction immune effect to induce sensitization and desensitization, but the molecular mechanisms of DEHP causing two-direction effect is certainly different.

### Serum OVA-IgE and serum T-IgE

As expected, a statistical difference of OVA-IgE was fund between saline and OVA control group (p<0.05) and an adjuvant effect was also fund in DEHP 3000 group compared with the saline control (p<0.05). In contrast it was unexpected that the levels of OVA-IgE in the OVA+DEHP exposure groups (OVA+DEHP30, OVA+DEHP300, OVA+DEHP3000) showed no statistically significant difference compared with the OVA exposure alone group (p>0.05). Although our results are in agreement with other related *in vivo* animal studies on adjuvant effect of DEHP [17], [20], [22], [24], [31], it seems contrary to an adjuvant effect of DEHP. One possible reason is that, as we know, the amount of IgE in the body is low, and in relation to T-IgE the fraction of specific OVA-IgE is even lesser, and a substantial amount exist bound on cell membranes of mast cells and basophils in the tissue. Therefor the OVA-IgE level remaining in circulating blood may be near to the limit to be detected by the ELISA kit. A clear circumstantial evidence for the above assumption is that the standard deviations of OVA-IgE measurements were relatively large, this phenomenon often results from poor measuring method but not from the real level of biomarker, and the inaccuracy of measuring method makes the statistic analysis with no significant difference hard to interpret.

It is very difficult to find studies from literature that has analysed serum T-IgE for characterizing an adjuvant effect of DEHP, the only one we found was carried out by Lee and his colleagues [26]. Their results (Fig. 5a of their paper) and our studies (Fig. 2a and Fig. 2b) are consistent: the serum T-IgE levels in antigen + DEHP exposure groups were significantly higher than that in antigen exposure alone group, and showing a dose-dependent pattern. The main difference between the two studies was that the antigen we used was OVA, while the antigen Lee used was keyhole limpet hemocyanin (KLH). The results of two studies suggest that serum T-IgE is an effective and sensitive biomarker reflecting adjuvant effects of DEHP. Moreover, according to the consistency of dose-response between serum T-IgE and lung histopathology, it is suggested that the increased T-IgE level may be an important pathological molecular event for DEHP-induced pulmonary inflammation of allergic asthma.

### The identified low-dose effect

Unfortunately, neither Lee's study nor our study had found the threshold (maximum non-effect dosage) for the serum T-IgE induced by DEHP in antigen co-exposure. Because in the two studies, even in the lowest exposure groups designed, 30 µg/kg bw/day (cumulative dose 1.53 mg DEHP/kg/51 d) in ours and 2 mg/kg bw/2 d (cumulative dose 14 mg DEHP/kg/21 d) in Lee (2004) the serum T-IgE still increased significantly, so more studies are needed. But the here presented data based on our experimental results, indicate that the lowest dose, 1.53 mg DEHP/kg/51 d, i.e 0.03 mg DEHP/kg/d, during prolonged exposure may have severe modulating effects on the immune system and thus be well above a risk level of exposure, which may be used for scientific reference.
